# Enantiomerically Pure Phosphonated Carbocyclic 2'-Oxa-3'-Azanucleosides: Synthesis and Biological Evaluation

**DOI:** 10.3390/molecules190914406

**Published:** 2014-09-12

**Authors:** Roberto Romeo, Caterina Carnovale, Salvatore V. Giofrè, Giulia Monciino, Maria A. Chiacchio, Claudia Sanfilippo, Beatrice Macchi

**Affiliations:** 1 Dipartimento Scienze del Farmaco e Prodotti per la Salute, Università di Messina, Via S.S. Annunziata, 98168 Messina, Italy; E-Mail: sgiofre@unime.it; 2 Dipartimento Scienze del Farmaco, Università di Catania, Viale A. Doria 6, 95125 Catania, Italy; E-Mails: giulimonci@hotmail.it (G.M.); ma.chiacchio@unict.it (M.A.C.); 3 Istituto di Chimica Biomolecolare del CNR, Via P. Gaifami 18, 95126 Catania, Italy; E-Mail: claudia.sanfilippo@icb.cnr.it; 4 Dipartimento di Medicina dei Sistemi, Università di Roma “Tor Vergata”, 00133 Roma, Italy; E-Mail: macchi@med.uniroma2.it

**Keywords:** PCOANs, *N*,*O*-modified nucleosides, antiretroviral

## Abstract

Starting from enantiomeric pure 1-[(3*S*,5*R*)- and 1-[(3*R*,5*S*)-3-(hydroxymethyl)-2-methylisoxazolidin-5-yl]-5-methylpyrimidine-2,4(1*H*,3*H*)-diones (−)**7a** and (+)**7b**, obtained by lipase-catalyzed resolution, pure diethyl{[(3*S*,5*R*)-2-methyl-5-(5-methyl-2,4-dioxo-3,4-dihydropyrimidin-1(2*H*)-yl)isoxazolidin-3-yl]methyl}phosphonate (−)**12a** and diethyl{[(3*R*,5*S*)-2-methyl-5-(5-methyl-2,4-dioxo-3,4-dihydropyrimidin-1(2*H*)-yl)isoxazolidin-3-yl]methyl}phosphonate (+)**12b** have been synthesized. The obtained compounds showed no cytotoxic activity *versus* the U937 cell line in comparison with AZT, and were poorly able to inhibit HIV infection *in vitro*.

## 1. Introduction

Continuous efforts in the development of new antiviral agents are a consequence of the urgent demand for new therapeutic agents in which improved biological activity against viruses is matched with low toxicity towards host cells. In this context, particular interest has been focused on the synthesis and the biological activity of nucleoside analogues, in which structural modifications of the heterocyclic bases and/or the sugar moiety of natural nucleosides have been performed [1,2,3,4,5,6,7,8,9,10,11,12,13]. A remarkable impulse for research on nucleoside analogues arose from the urgent need to find a therapeutic approach to combat human immunodeficiency virus (HIV) infections. In the eighties, research on antiretrovirals was very fruitful and in 1986 the *in vitro* anti-HIV activity of the prototype antiretroviral drug, 3'-azido-3'-deoxythymidine (AZT, zidovudine) was demonstrated by Mitsuya and Broder [14,15].

**Figure 1:**
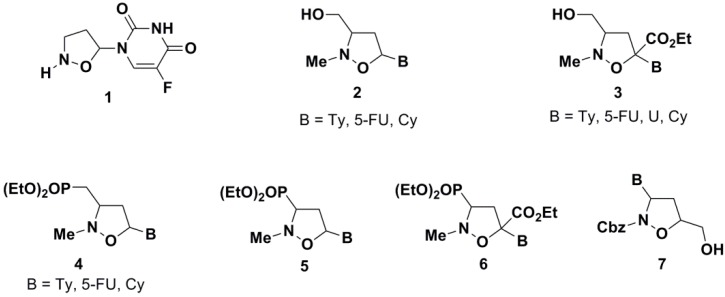
*N*,*O*-modified nucleosides.

Since the discovery of AZT, a number of nucleoside analogues have been designed, sharing structural similarities with each other and mimicking endogenous nucleosides. In this context, modified nucleosides, in which alternative carbon or heterocyclic systems replaces the furanose ring, have attracted special interest by virtue of their biological action as antiviral or anticancer agents [16,17,18,19,20,21,22,23,24,25]. Among these, *N*,*O*-nucleosides **1**–**7**, characterized by the presence of an isoxazolidine moiety (carbocyclic-2'-oxo-3'-azanucleosides), have emerged as an interesting class of dideoxynucleoside analogues endowed with potential pharmacological activity (Figure 1) [26,27,28,29,30,31,32]. These compounds, which mimic natural nucleosides, exert their antiviral activity by competitive reversible inhibition of reverse transcriptase, acting as viral DNA chain terminators, or they behave as antimetabolites, competing with physiological nucleosides and consequently interacting with a large number of intracellular targets to induce cytotoxicity.

The biological activity of nucleoside analogues (NA) showing antiviral properties is strictly linked to their conversion, through cellular enzymes, to the corresponding biologically active triphosphate form, which interacts with viral RT or interferes with cell growth, slowing the cell cycle progression. One of the metabolic drawbacks of nucleoside analogues (NA) is the retention of their stability following the triphosphorylation inside the host cell. To overcome the instability of triphosphate nucleic acid (NA), several strategies have been proposed to increase their resistance toward phosphohydrolases or to ensure a more efficient phosphorylation within the target cells [33,34,35,36,37,38,39,40,41,42,43,44,45,46].

Instability of the phosphate forms of nucleoside analogues (NA) has been, at least partially, overcome by the introduction in their molecular structures of phosphonate groups. With the aim of bypassing the first limiting step of phosphorylation [47,48,49], we have synthesized a series of racemic phosphonated *N*,*O*-nucleosides (PCOANs) **4**, as mimics of monophosphate nucleosides [29,30,31], by exploitation of the 1,3-dipolar cycloaddition methodology [50,51], starting from nitrones containing a phosphonic group.

PCOANs **4** show low levels of cytotoxicity and exert a specific inhibitor activity on two different RT: these compounds have been proposed to ensure long lasting control of HTLV-1, an oncogen retrovirus associated with adult leukemia/limphoma (ATLL) and with myelopathy, tropical spastic paraparesis. By considering the epidemiological and pathogenetic relevance of HIV infection and that HTLV-1 and HIV are both lymphotropic retroviruses, we decided to investigate the effect of PCOANs on an *in vitro* model of HIV transmission.

Both enantiomeric purity and absolute configuration could be key factors in determining the physiological activity of a drug [52]; thus, in this paper, we have investigated the development of an efficient approach to obtain PCOANs in enantiomerically pure form, in order to test the biological activity of pure enantiomers.

## 2. Results and Discussion

**Scheme 1:**
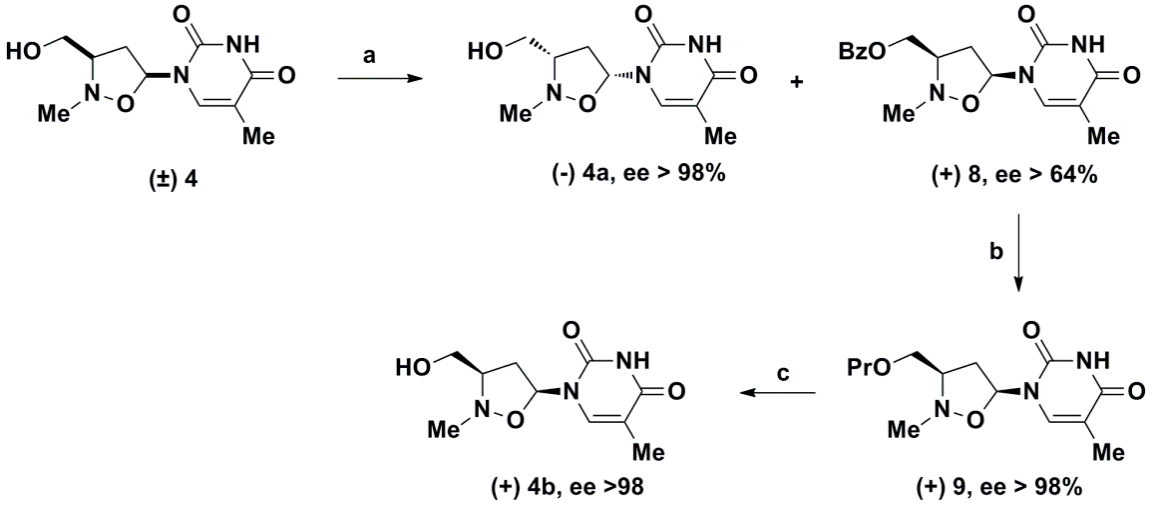
Synthesis of racemic *N*,*O*-thymine nucleoside and its double enzymatic resolution.

**Scheme 2:**
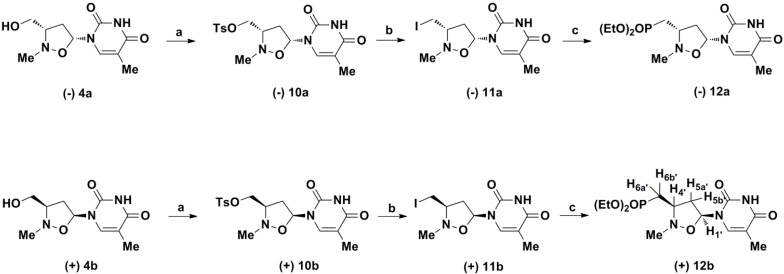
Synthesis of enantiopure phosphonated *N*,*O*-thymine nucleoside.

Our synthetic approach to the pure enantiomers diethyl{[(3*S*,5*R*)-2-methyl-5-(5-methyl-2,4-dioxo-3,4-dihydropyrimidin-1(2*H*)-yl)isoxazolidin-3-yl]methyl}phosphonate (**12a**) and diethyl-{[(3*R*,5*S*)-2-methyl-5-(5-methyl-2,4-dioxo-3,4-dihydropyrimidin-1(2*H*)-yl)isoxazolidin-3-yl]methyl}phosphonate (**12b**) is described in Scheme 1 and Scheme 2.

*Reagents and conditions*: (a) Lipozyme, 1,4-dioxane, vinyl benzoate, 45 °C; (b) NH_4_OH/MeOH, 50 °C, 8 h, lipozyme, vinyl propionate, 45 °C; (c) NH_4_OH/MeOH, 50 °C, 8 h.

Thus, the racemic *N*,*O*-nucleoside isoxazolidine **4**, obtained by 1,3-dipolar cycloaddition reaction of C-[(tert-butyldiphenylsilyl)oxy]-N-methylnitrone with vinyl acetate, followed by Hilbert–Jones nucleosidation with silylated thymine and TBAF treatment, was converted into the enantiomeric pure 1-[(3*S*,5*R*)-3-(hydroxymethyl)-2-methylisoxazolidin-5-yl]-5-methylpyrimidine-2,4(1*H*,3*H*)-dione (−)**4a** and 1-[(3*R*,5*S*)-3-(hydroxymethyl)-2-methylisoxazolidin-5-yl]-5-methylpyrimidine-2,4(1*H*,3*H*)-dione (+)**4b** by lipase-catalyzed resolution in 1,4-dioxane [53].

*Reagents and conditions*: (a) Tosyl chloride, TEA, CH_2_Cl_2_, rt, 24 h; (b) NaI, acetone, reflux, 72 h; (c) P(OEt)_3_, 180 °C, 16 h.

The subsequent reaction of (−)**4a** with tosyl chloride affords the corresponding tosylate (−)**10a**, which was transformed into the 2-methyl-5-(5-methyl-2,4-dioxo-3,4-dihydropyrimidin-1(2*H*)-yl)isoxazolidin-3-yl]methyl}phosphonate (−)**12a** by reaction with NaI in acetone, followed by triethylphosphite treatment (Scheme 2).

Analogously, compound (+)**4b** was converted in (+)**12b** by similar reactions. HPLC chromatography showed the absence of any diasteromeric product, which could be originated from the sequence of the reaction. NOE measurements confirm that the configurations of the reagents is maintained in the obtained products.

**Figure 2:**
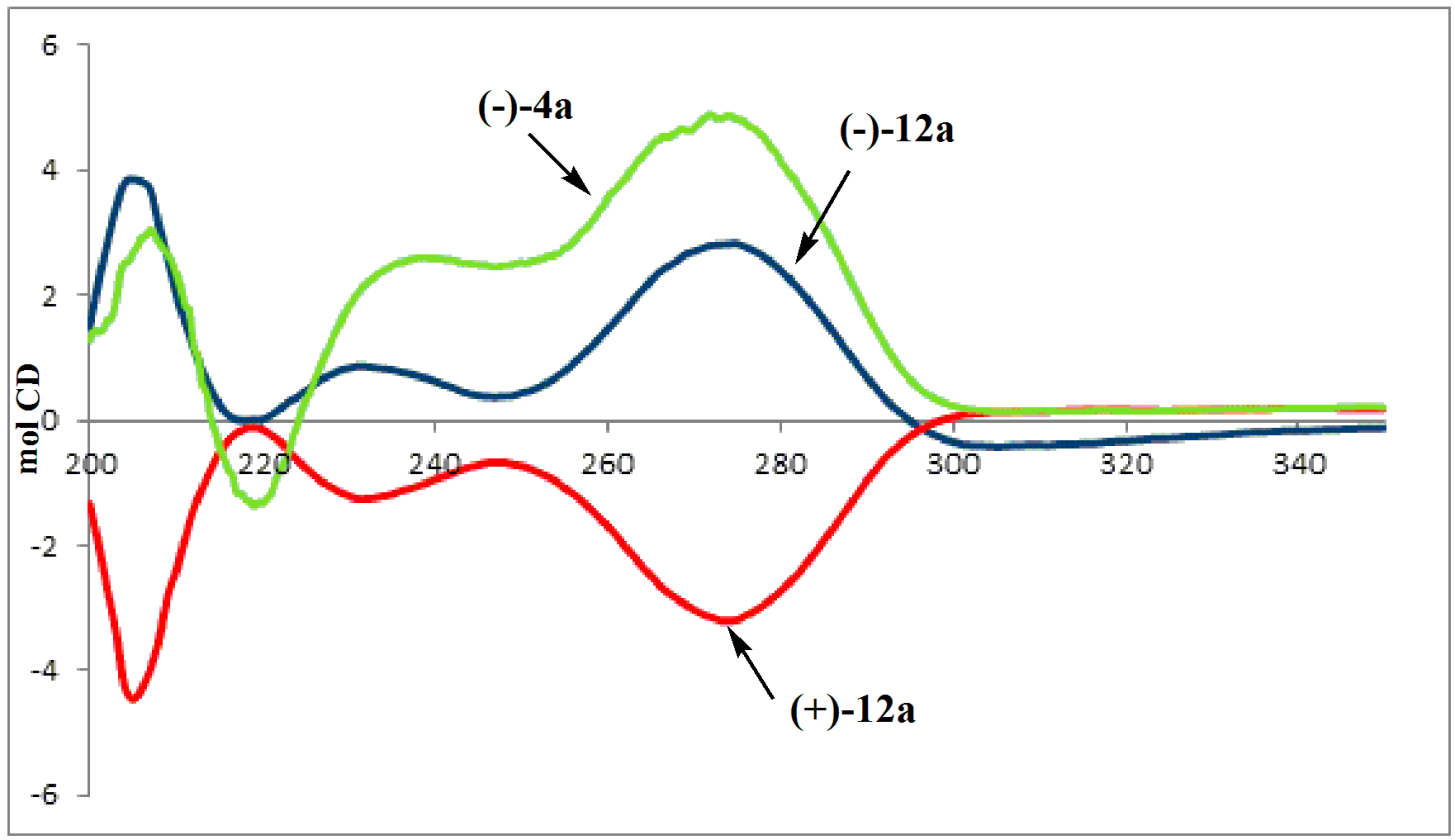
CD Spectra recorded in methanol of (−)**4a**, (−)**12a** and (+)**12b**.

The absolute configuration of (3*S*,5*R*) was assigned to compound (−)**12a** according to its origin from (−)4a in a reaction pathway which does not involve any change to the stereogenic centers. CD spectra, reported in Figure 2, confirm this assumption: the CD spectrum obtained for (−)**12a** was found to be a good approximation matching that registered for (3*S*,5*R*) (−)**4a**, thus allowing the assignment of the same absolute configuration to the stereogenic centers of both compounds. Analogous data confirm as (3*R*,5*S*) the absolute configuration of (+)**12b**.

The biological activity of enantiomers (−)**12a** and (−)**12b** was tested by assessing their cytotoxic effect towards a monocytoid human cell line, U937, and their specific activity on HIV infection *in vitro*. 3'-Azido-2',3'-dideoxythymidine (AZT) was used as positive control, since it is a prototype of nucleoside analogs (NRTI) acting as chain terminator and endowed with potent anti-HIV activity *in vitro.* The obtained results indicated that both enantiomers **12** showed no cytotoxicity *versus* the U937 cell line in comparison with AZT, which exhibited a IC_50_ of 800 µM. On the other hand, the compounds were poorly able to inhibit HIV infection *in vitro*, showing a IC_50_ > of 1000 µM with respect to AZT. Although the tested molecules showed poor specific anti-HIV activity, their low toxicity encourages us to continue search for cytostatic compounds in the phosphonated *N*,*O*-nucleoside class with special attention to different heterocyclic systems as mimics of natural nucleobases.

## 3. Experimental Section

### 3.1. General Information

Solvents and reagents were used as received from commercial sources. Melting points were determined with a Kofler apparatus. Thin layer chromatographic separations were performed on Merck silica gel 60-F254 precoated aluminum plates (Merk, Darmstadt, Germany). Flash chromatography was accomplished on Merck silica gel (200–400 mesh). Preparative separations were carried out by a Buchi C-601MPLC (BUCHI Italia S.r.l., Milano, Italy), using Merck silica gel 0.040–0.063 mm and the eluting solvents were delivered by a pump at the flow rate of 3.5–7.0 mL/min. HRMS were determined with a TSQ Quantum XLS Triple Quadrupole GC-MS/MS (Thermo Scientific, Waltham, MA, USA). ^1^H-NMR (500 MHz) and ^13^C-NMR (125 MHz) spectra were recorded in CDCl_3_, on a Varian 500 instrument (Agilent Technologies, Palo Alto, CA, USA). Chemical shifts (δ) are reported in ppm relative to TMS and coupling constants (*J*) in Hz. CD spectra 3 were registered at 20 °C in methanol (0.1 cm cell length) on a JASCO J-810 spectropolarimeter (JASCO, Europe S.r.l., Lecco, Italy).

### 3.2. Synthesis of ((3S,5R)-5-(3,4-Dihydro-5-methyl-2,4-dioxopyrimidin-1(2H)-yl)-2-methylisoxa-zolidin- 3-yl)methyl 4-methylbenzenesulphonate (−);)**10a**

To a solution of compound (−)**4a** (0.400 g, 1.6 mmol) in CH_2_Cl_2_ (20 mL), Et_3_N (0.25 mL, 1.8 mmol) was added. TsCl (0.349 g, 1.8 mmol) was then added slowly at 0 °C and the reaction mixture was stirred at room temperature for 24 h. The solvent was removed under vacuum and the residue was purified by MPLC, using CH_2_Cl_2_/MeOH 99:1 as eluent, to give (−)**10a** in 70% yield (0.44 g). Compound (−)**10a** was recrystallized from hexane. White solid, m.p. 134–136 °C, 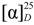 = −84.9 (*c* 0.25, MeOH). ^1^H-NMR (CDCl_3_): δ = 1.77 (s, 3H); 1.93–2.04 (m, 1H), 2.32 (s, 3H); 2.63 (s, 3H); 2.77–2.91 (m, 2H); 3.89–4.07 (m, 2H), 5.94–6.02 (m, 1H), 7.23 (d, *J* = 8.2 Hz, 2H), 7.46 (s, 1H); 7.63 (d, *J* = 8.2 Hz, 2H), 9.37 (bs, 1H). ^13^C-NMR (CDCl_3_): δ = 8.54, 17.48, 36.53, 40.26, 61.81, 63.64, 89.98, 106.58, 123.67, 125.90, 131.73, 138.66, 144.09, 151.95, 160.09. HRMS: calcd for C_17_H_21_N_3_O_6_SNa^+^ 418.1043, found 418.1050.

### 3.3. Synthesis of (3R,5S)-1-(3-(Iodomethyl)-2-methylisoxazolidin-5-yl)-5-methylpyrimidin-2,4-(1H,3H) dione (−)**11a**

Compound (−)**10a** (0.32 g, 0.81 mmol) was added to a solution of NaI (0.600g, 4 mmol) in acetone (20 mL) and the reaction mixture was refluxed for 24 h. After cooling, acetone was removed *in vacuo* and chloroform was added to the residue. The resulting organic phase was filtered and evaporated. The residue was purified by MPLC, using CH_2_Cl_2_: MeOH 99:1 as eluent, to give the compound (−)**11a** in 93% yield (0.260 g). Compound (−)**11a** was recrystallized from hexane. Pale yellow solid; m.p. 157–159 °C. 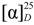 = −88.50 (*c* 0.5, MeOH); ^1^H-NMR (CDCl_3_): δ = 1.96 (s, 3H), 2.20 (ddd, *J* = 13.7, 9.2, 4.1 Hz, 1H), 2.51–2.64 (m, 1H), 2.70 (s, 3H), 3.06 (m, 1H), 3.11 (dd, *J =* 10.7, 4.1Hz, 1H), 3.25 (dd, *J* = 10.7, 3.2 Hz, 1H), 6.16 (dd, *J* = 7.8, 4.1 Hz, 1H), 7.76 (s, 1H), 8.54 (bs, 1H). ^13^C-NMR (CDCl_3_): δ = 8.44; 25.45, 38.99, 41.18, 63.28, 77.44, 110.20, 132.30, 150.20, 163.4. HRMS: calcd for C_10_H_14_N_3_O_3_INa^+^ 373.9972, found 373.9981

### 3.4. Synthesis of Diethyl{(1'R,4'S)-1'-[[(5-methyl-2,4-dioxo-3,4-dihydropyrimidin-1(2H)-yl]-3'-methyl-2'-oxa-3'-aza-cyclopent-4'-yl]}methylphosphonate (−)**12a**

Compound (−)**12a** was recrystallized from hexane. White solid, m.p. 134–136 °C. 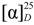 = −83.4 (*c* 0.50, MeOH); ^1^H-NMR (CDCl_3_): δ= 1.30 (dt, 6H, *J =* 3.6 and 7.1 Hz), 1.89 (ddd, 1H, *J =* 10.2, 15.0 and 18.3 Hz, H_5'a_), 1.93 (d, 3H, *J =* 1.3Hz), 2.08 (ddd, 1H, *J* = 3.2, 15.0 and 20.6 Hz, H_5'b_), 2.23 (ddd, 1H,*J* = 4.6, 10.1 and 13.8 Hz, H_6'a_), 2.75 (s, 3H, N-CH_3_), 2.98 (dddd, 1H, *J =* 3.2, 7.0, 10.1 and 10.2 Hz, H_4'_), 3.18 (ddd, 1H, *J =* 7.0, 7.9 and 13.8 Hz, , H_6'b_), 4.10–4.17 (m, 4H), 6.20 (dd, 1H, *J =* 4.6 and 7.9 Hz, H_1'_), 7.66 (q, 1H, *J =* 1.3 Hz, H_6_), 9.56 (bs, 1H, NH); ^13^C-NMR (CDCl_3_); δ: 12.57, 16.33, 16.38, 27.88 (d, *J =* 143.2 Hz), 42.68, 44.35, 61.93, 61.98, 63.37, 81.94, 110.66, 135.96, 150.56, 164.11 CD: λ_ext_ 200 (Δε +1.49), 206 (Δε +3.82), 219 (Δε −0.11), 233(Δε +0.84), 249 (Δε +0.38), 275 (Δε +2.82), 290 (Δε +0.67), 307 (Δε −0.42), 360 (Δε −0.08).

An analogous reaction pathway, starting from (+)**4b**, afforded enantiomerically pure (+)**10b**, (+)**11b** and (+)**12b**. CD spectra of (+)**12b**: λ_ext_ 200 (Δε −1.33), 205 (Δε −4.47), 219 (Δε −0.14), 233(Δε −1.24), 249 (Δε −0.70), 275 (Δε −3.19), 290 (Δε −0.96), 305 (Δε +0.16), 360 (Δε +0.20).

### 3.5. Biological Assay

HIV infection was carried on by using a stable T cell line (CEM) containing a plasmid encoding a green fluorescence protein (GFP) driven by the HIV-1 long terminal repeat [54]. Infection was carried on as previously b shown with some modification [55]. Briefly, 5 × 10^5^ CEM-GFP were infected with a volume of supernatant from HIV chronically infected H9 cells equivalent to 20 ng/mL of HIV p24, for 2 h in 100 µL in DMSO in presence of 1000, 100, 10 and 1 µM concentration of compounds. Then medium was added and the cultures were incubated for 72 h. The inhibition was assessed on the basis of GFP expression in the different culture conditions. Cytotoxicity assays were performed by MTS assay kit (Promega Corporation, Madison, WI, USA). Briefly Inhibition of cell metabolic activity revealed by reduction of the oxidative burst was detected through formazan product formation, using a commercial colorimetric kit (MTS [3,4-(5-dimethylthiazol-2-yl)-5-(3-carboxymethoxyphenyl)-2-(4-sulfophenyl)-2*H*-tetrazolium salt]) (Cell Titer 96 Aqueous One Solution; Promega). The assay was performed by seeding 1 × 10^4^ U937 cells in the presence or absence of the different compounds at concentrations ranging from 1000 to 1 µM.

## 4. Conclusions

In this work, an efficient synthetic pathway towards diethyl{[(3*S*,5*R*)-2-methyl-5-(5-methyl-2,4-dioxo-3,4-dihydropyrimidin-1(2*H*)-yl)isoxazolidin-3-yl]methyl}-phosphonate (**12a**) and diethyl{[(3*R*,5*S*)2-methyl-5-(5-methyl-2,4-dioxo-3,4-dihydro-pyrimidin-1(2*H*)-yl)isoxazolidin-3-yl]methyl}phosphonate (**12b**) has been developed, starting from enantiomerically pure 1-[(3*S*,5*R*)-3-(hydroxymethyl)-2-methylisoxazolidin-5-yl]-5-methylpyrimidine-2,4(1*H*,3*H*)-dione ((−)**4a**) and 1-[(3*R*,5*S*)-3-(hydroxymethyl)-2-methylisoxazolidin-5-yl]-5-methylpyrimidine-2,4(1*H*,3*H*)-dione ((+)**4b**) by tosylation, iodination and Arbuzov reaction. The obtained compounds show no cytotoxicity *versus* the U937 cell line in comparison with AZT, but they were found to be poorly able to inhibit HIV infection *in vitro*.
